# Imaging and pathological characteristics of hepatosplenic EBV-positive inflammatory follicular dendritic cell sarcoma

**DOI:** 10.3389/fonc.2025.1552193

**Published:** 2025-05-16

**Authors:** Yang-Yang Chen, Peng-Ming Wang, Li-Zhu Deng, Pei-Heng Li, Chen-Guang Li, Yu-Hua He, Feng Chen, Jun-Yan Yue

**Affiliations:** ^1^ Department of Radiology, The First Affiliated Hospital of Xinxiang Medical University, Xinxiang, China; ^2^ Department of Radiology, Zhongshan Hospital of Xiamen University, Xiamen, China; ^3^ Department of Radiology, The First Affiliated Hospital, School of Medicine, Zhejiang University, Hangzhou, China

**Keywords:** EBV-positive, imaging features, inflammatory follicular dendritic cell sarcoma, liver, spleen

## Abstract

**Background:**

The aim of this study is to explore the CT and MRI characteristics of hepatosplenic Epstein-Barr virus (EBV)-positive inflammatory follicular dendritic cell sarcoma (IFDCS) and to correlate these findings with pathological characteristics to enhance diagnostic understanding.

**Case description:**

A retrospective analysis was conducted on 16 patients with surgically confirmed hepatosplenic EBV-positive IFDCS, who underwent CT and MRI between January 2015 and May 2024. Clinical, pathological, and imaging data were evaluated.

**Results:**

Hepatosplenic EBV-positive IFDCS primarily occurred in middle-aged adults, with a greater prevalence in females. All cases presented as solitary, well-defined masses, either round or oval in shape. Calcification was noted in one case, while necrosis and cystic degeneration were observed in nine cases. Contrast-enhanced CT and MRI showed that 15 cases exhibited heterogeneous, mild to moderate persistent uneven enhancement of the tumor parenchyma, with most lesions appearing hypodense or hypointense in the delayed phase. The majority of cases showed a hypodense or hypointense capsule with delayed enhancement. Solid tumor components appeared hyperintense on diffusion-weighted imaging (DWI), with apparent diffusion coefficient values similar to those of the spleen. One case of hepatic EBV-positive IFDCS exhibited an atypical enhancement pattern characterized by rapid wash-in and slow wash-out.

**Conclusion:**

MRI proved superior in visualizing the tumor capsule and providing qualitative diagnostic information, yielding crucial information for clinical diagnosis and preoperative evaluation.

## 1 Introduction

Follicular dendritic cell sarcoma (FDCS) is an uncommon malignant tumor that may affect lymph nodes and extranodal sites, usually presenting aggressively in the abdominal region (1). Clinically, EBV+ IFDCS is characterized by female predominance and higher prevalence among Asian populations. Most patients are asymptomatic, with lesions typically discovered incidentally during routine examinations. Monda et al. first documented four cases of tumors arising from follicular dendritic cells in 1986. In 2001, Cheuk et al. identified inflammatory pseudotumor-like follicular dendritic cell sarcoma as an EBV-associated variant of follicular dendritic cell origin (2, 3). In the fifth edition (2022) of the WHO classification of lymphoid and hematopoietic neoplasms, this entity has been classified under tumors of lymphoid stromal origin as EBV-positive inflammatory follicular dendritic cell sarcoma (EBV+ IFDCS) (4).

Hepatic and splenic EBV+ IFDCS exhibits relatively indolent biological behavior and low-grade malignancy, primarily affecting the spleen, liver, or both organs simultaneously. Case reports have also documented its occurrence in other extranodal sites, including the lungs, gastrointestinal tract, and pancreas (3, 5). The clinical presentation of EBV+ IFDCS is non-specific, contributing to its rarity and a correspondingly high rate of misdiagnosis. In clinical practice, the authors have noted that the diffusion-weighted imaging (DWI) signal and the apparent diffusion coefficient (ADC) map of EBV+ IFDCS lesions are similar to those of the adjacent spleen tissue. However, existing literature lacks reports correlating this disease with splenic MRI features, especially with regard to DWI and ADC characteristics.

In this study, clinical, pathological, and imaging data from 16 cases of surgically confirmed hepatic and splenic EBV+ IFDCS were collected from The First Affiliated Hospital of Xinxiang Medical University, The First Affiliated Hospital of Zhejiang University School of Medicine, and Zhongshan Hospital Xiamen University. This analysis aims to enhance the understanding of this rare condition and improve diagnostic accuracy through better characterization of its imaging features.

## 2 Materials and methods

### 2.1 General information

A retrospective analysis was conducted on 16 patients diagnosed with surgically confirmed splenic EBV+ IFDCS between January 2015 and May 2024. The following data were recorded: patient gender, age, lesion location, clinical manifestations, treatment course, and follow-up. The inclusion criteria for this study were: (1) Histopathological confirmation of EBV+ IFDCS following surgery; (2) Availability of preoperative non-contrast and contrast-enhanced CT or MRI; (3) No relevant treatment received prior to the imaging examination. The exclusion criteria were as follows: (1) Incomplete clinical or pathological data; (2) Presence of other malignant tumors or severe comorbidities.

### 2.2 Methods

Among the 16 patients with hepatosplenic EBV+ IFDCS included in this study, 14 patients underwent CT examinations (12 with splenic EBV+ IFDCS and 2 with hepatic EBV+ IFDCS), and 12 patients underwent MRI examinations (9 with splenic EBV+ IFDCS and 3 with hepatic EBV+ IFDCS). A total of 10 patients received both CT and MRI examinations.

#### 2.2.1 CT examination method

The CT examinations were conducted using GE Discovery CT750HD and GE Revolution 256-slice CT scanners. The scanning range extended from the diaphragm to the inferior margin of the spleen. Both non-contrast (without contrast material administration) and contrast-enhanced scans were performed. Scanning parameters included a slice thickness of 0.625 mm, a tube voltage of 120 kV, and a tube current between 350 and 450 mA, with images reconstructed using a 512 × 512 matrix. Contrast materials as administered via the median cubital vein, using Iohexol with an iodine concentration of 300 mg/mL at a flow rate of 4.0 mL/s. Contract-enhanced imaging was performed in three phases: arterial phase (25 to 30 seconds post-injection), portal venous phase (60 to 70 seconds post-injection), and delayed phase (85 to 90 seconds post-injection).

#### 2.2.2 MRI examination method

MRI was performed using GE Signa HDxt 3.0T MR and Philips Ingenia CX 3.0T MR scanners. Enhanced imaging was acquired using the Liver acquisition with volume acceleration (LAVA) sequence, with a slice thickness of 5 mm, a matrix resolution of 320 × 256, a repetition time (TR) of 3.3 ms, an echo time (TE) of 1.5 ms, and a flip angle of 10°. Contrast material was administered via high-pressure injection into the antecubital vein. Gadopentetate dimeglumine was used as the contrast agent, delivered at a rate of 2.0 to 3.0 mL/s, with a dose of 0.1 mmol/kg. Enhanced imaging was performed in multiple phases: early arterial phase (15 to 20 seconds), late arterial/early portal venous phase (40 to 55 seconds), late portal venous phase (140 to 180 seconds), and delayed phase (300 to 360 seconds).

#### 2.2.3 Image analysis method

The CT and MRI characteristics of the lesions were independently assessed by two senior radiologists (with 15 and 12 years of experience, respectively) specializing in chest and abdominal imaging. CT image analysis included an assessment of lesion size, morphology, density, presence of calcification, enhancement patterns, and degree of enhancement. MRI analysis included the evaluation of lesion location, size, shape, margins, signal intensity on non-contrast scans, and enhancement characteristics. The DWI signal characteristics of EBV+ IFDCS lesions were compared to those of the spleen. ADC values for the solid components of the lesions and normal spleen tissue were measured on ADC maps. In the event of disagreement, a consensus was reached through consultation with an associate chief physician.

#### 2.2.4 Pathological examination method

Gross specimens from all 16 cases were paraffin-embedded, sectioned, and subjected to H&E staining, along with immunohistochemical staining for examination under an optical microscope. All 16 specimens also underwent *in situ* hybridization tests for Epstein-Barr virus-encoded small RNA (EBER) detection, using oligonucleotide probes to confirm EBV association.

### 2.3 Statistical analysis

Data analysis was conducted using SPSS version 27.0 software. Inter-group comparisons were carried out using the independent samples *t*-test. Continuous variables were assessed for normality of distribution. Normally distributed continuous variables are presented as mean ± standard deviation (x¯ ± s). Non-normally distributed measurement data are expressed as median (M (P25, P75)*)* and analyzed using the Mann-Whitney *U* test. A *P* < 0.05 was considered statistically significant.

## 3 Results

### 3.1 General information

Among the 16 patients, 15 were initially detected during routine physical examinations (when imaging revealed liver or splenic masses), while 1 case of splenic EBV+ IFDCS presented with hematochezia. All cases were subsequently confirmed by surgical pathology. Tumor markers were not elevated in any of the 16 patients. The general information of the 16 patients with EBV+ IFDCS is presented in **Table 1**.

**Table 1:** Demographic and Clinical Characteristics of 16 Patients with EBV+ IFDCS.

Variables	Splenic EBV+ IFDCS	Hepatic EBV+ IFDCS	*P* value
Mean age (years)	62±9	45±25	0.360
Median age (years)	60	57	
Age range (years)	44~76	16~62	
Mean diameter (cm)	6.2±4.0	3.5±2.3	0.299
Median diameter (cm)	4.9	2.5	
Diameter range (cm)	3.1~19.0	1.9~6.2	
Follow-up time (months)	1~60	2~12	

### 3.2 Imaging findings

All 16 cases in this study presented as solitary lesions, appearing as well-defined elliptical or round masses. Among these, 9 cases displayed necrosis or cystic degeneration within the tumors, 1 case was associated with hemorrhage, and 1 case exhibited calcification. Detailed imaging characteristics are summarized in **Table 2**.

**Table 2:** Imaging characteristics of 16 cases of hepatic and splenic EBV+ IFDCSs.

Diagnostic Methods	Splenic EBV+ IFDCS (n = 13)		Hepatic EBV+ IFDCS (n = 3)	
CT	Non-contrast scan (n = 12)		Non-contrast scan (n = 2)	
	Isodense	3 (25%)	Isodense	1 (50%)
	Hypodense	8 (67%)	Hypodense	1 (50%)
	Mixed high and low density	1 (8%)	Mixed high and low density	0
	Enhancement pattern (n = 12)		Enhancement pattern (n = 12)	
	Fill-in enhancement	12 (100%)	Fill-in enhancement	1 (50%)
	Rapid wash-in and slow wash-out pattern	0 (0%)	Rapid wash-in and slow wash-out pattern	1 (50%)
	Delayed phase (n = 12)		Delayed phase (n = 12)	
	Hypodense	11 (92%)	Hypodense	1 (50%)
	Isodense	1 (8%)	Slightly hyperdense	1 (50%)
MRI	Non-contrast scan (n = 9)		Non-contrast scan (n = 3)	
	T1 signal		T1 signal	
	Slightly hyperintense	2 (22%)	Slightly hyperintense	1 (33%)
	Isointense	3 (34%)	Isointense	0 (0%)
	Slightly hypointense	4 (44%)	Slightly hypointense	2 (67%)
	T2 signal		T2 signal	
	Slightly hyperintense	9 (100%)	Slightly hyperintense	3 (100%)
	Enhancement pattern (n = 9)		Enhancement pattern (n = 3)	
	Fill-in enhancement	9 (100%)	Fill-in enhancement	2 (67%)
	Rapid wash-in and slow wash-out pattern	0 (0%)	Rapid wash-in and slow wash-out pattern	1 (33%)
	Delayed phase (n = 9)		Delayed phase (n = 3)	
	Hypointense	5 (56%)	Slightly hyperintense	2 (67%)
	Isointense	4 (44%)	Slightly hypointense	1 (33%)
Capsule visualization	CT (n=12)	7 (58%)	CT (n=2)	1/2 (50%)
	MRI (n=9)	8 (89%)	MRI (n=3)	2/3 (67%)
Cystic degeneration, necrosis visualization	CT (n=12)	8 (67%)	CT (n=2)	1/2 (50%)
	MRI (n=9)	7 (78%)	MRI (n=3)	1/3 (33%)
Enhancement pattern of cystic degeneration, necrosis areas	CT (n=8)		CT (n=1)	
	No change in area	4 (50%)	Gradually decreasing area	1 (100%)
	Gradually decreasing area	4 (50%)		
	MRI (n = 7)		MRI (n = 1)	
	No change in area	1 (14%)	Delayed enhancement	1 (100%)
	Gradually decreasing area	5 (72%)		
	Delayed enhancement	1 (14%)		

#### 3.2.1 Imaging findings of splenic EBV+ IFDCS

**Figure 1:**
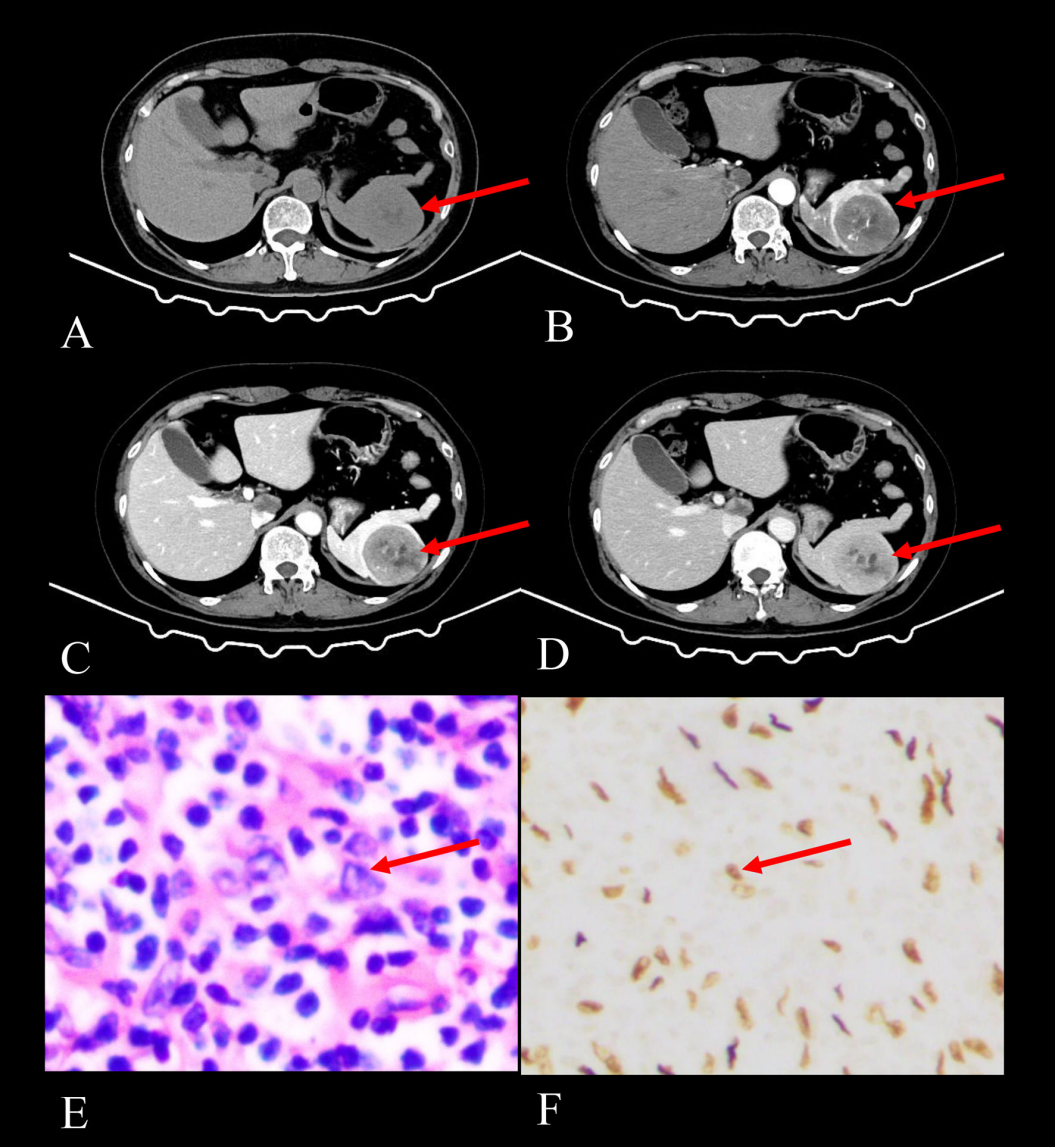
Imaging and histopathological findings of a 52-year-old female with splenic EBV+ IFDCS detected during a physical examination 2 months prior. **(A)** Axial CT non-contrast scan shows a solitary heterogeneous soft tissue mass in the spleen (arrow), exhibiting iso- to hypodensity. **(B)** Axial CT enhanced scan in the arterial phase shows heterogeneous enhancement with patchy and punctate areas of high enhancement, demonstrating disorganized vascular shadows (arrow). **(C)** Axial CT enhanced scan in the venous phase reveals increased enhancement of the lesion with a filled-in appearance and visible hypodense capsule (arrow). **(D)** Axial CT enhanced scan in the delayed phase indicates decreased enhancement compared to the venous phase, with persistent visualization of the capsule (arrow) and non-enhancing necrotic areas progressively shrinking. **(E)** HE staining (400× magnification) shows tumor cells that are spindle-shaped or polygonal (arrow), featuring large and irregular nuclei, amidst a background rich in lymphocytes and plasma cells. **(F)** EBER staining (400× magnification) reveals positive EBER expression in the tumor cells (arrow), confirming EBV infection.

CT Findings: Among the 13 patients with splenic EBV+ IFDCS, 12 underwent both non-contrast and contrast-enhanced CT examinations. On non-contrast scans, the lesions appeared iso- or slightly hypodense compared to the surrounding splenic tissue. Eight cases (67%) exhibited necrosis or cystic degeneration, while 1 case was complicated by hemorrhage. Additionally, 4 cases (33%) without necrosis or cystic degeneration had tumor diameters less than 5 cm. One tumor displayed peripheral encapsulated calcification. The tumors exhibited a hypodense capsule at the margin in 8 cases (67%). During contract enhancement, all tumors displayed a heterogeneous enhancement pattern in their solid components. In the delayed phase, the solid components appeared hypodense in 11 cases and isodense in 1 case. The areas of central necrosis or cystic degeneration showed no change in size in 4 cases and gradually decreased in size in 4 cases. Furthermore, disorganized vascular shadows were observed in the solid components during the arterial phase in 4 cases (as depicted in **Figure 1**, arrow).

MRI findings: Among the 13 patients with splenic EBV+ IFDCS, 9 underwent both non-contrast and contrast-enhanced MRI examinations. On T1-weighted imaging (T1WI), 2 cases exhibited slightly hyperintense signals, 3 cases showed isointense signals, and 4 cases shown slightly low signals. On T2-weighted imaging (T2WI), all cases revealed homogeneous or heterogeneous slightly hyperintense signals, with a heterogeneous enhanced filling-in pattern observed on contrast-enhanced images. Of these cases, 7 (78%) displayed areas of necrosis or cystic degeneration, which appeared as slightly hypointense signals on T1WI and slightly hyperintense signals on T2WI.

In the delayed phase, 5 cases showed low signal intensity tumors, while 4 cases presented isointense signals. In 1 case, the areas of cystic degeneration and necrosis exhibited enhancement in the delayed phase. Additionally, in 5 cases, the non-enhancing areas of cystic degeneration and necrosis gradually decreased in size, while in 1 case, these areas remained unchanged across all phases. The majority of cases (8, or 89%) demonstrated a ring-like hypointense capsule on T2WI sequences, with capsule enhancement noted in the delayed phase. No fat signals were detected within the tumors. On DWI, the solid components showed high signal intensity, whereas on ADC maps, they appeared as low signal intensity.

#### 3.2.2 Imaging findings of hepatic EBV+ IFDCS

Hepatic findings: Three cases of hepatic EBV+ IFDCS were reported, with 2 cases undergoing both non-contrast and contrast-enhanced CT examinations. On non-contrast scans, these lesions appeared iso- or slightly hypodense compared to the surrounding liver tissue. In one case (diameter 6.2 cm), necrosis or cystic degeneration was present within the tumor, accompanies by a hypodense capsule. Two enhancement patterns were observed: (1) Heterogeneous enhanced filling-in pattern: In the case with a 6.2 cm diameter tumor, the solid components displayed a heterogeneous enhancement pattern, remaining hypodense in the delayed phase. (2) Rapid wash-in and slow wash-out pattern: In the case with a 1.9 cm diameter tumor, significant enhancement was noted in the arterial phase, with a decrease in enhancement observed in the venous and delayed phases, yet although the density remained slightly elevated compared to the adjacent liver parenchyma.

**Figure 2:**
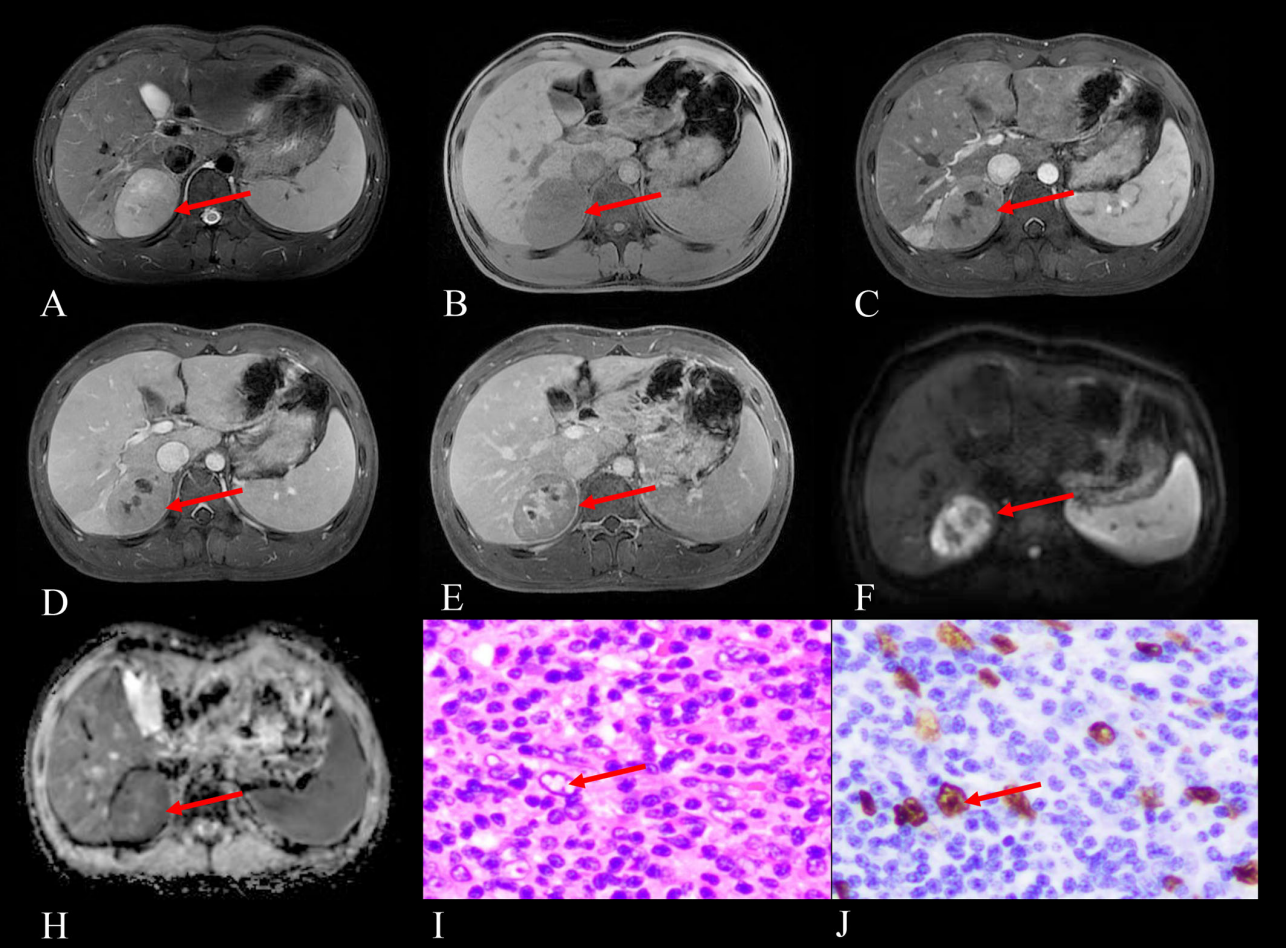
Imaging and histopathological findings of a 16-year-old male with EBV+ IFDCS in the right posterior lobe of the liver, identified during a physical examination half a month prior. **(A)** T2WI reveals heterogeneous hyperintensity in the tumor (arrow). **(B)** Non-enhanced T1WI shows a heterogeneous, slightly hypointense signal mass (arrow). **(C)** Arterial phase contrast-enhanced T1WI demonstrates mild heterogeneous enhancement of the tumor (arrow). **(D)** Portal venous phase contrast-enhanced T1WI shows progressive fill-in enhancement pattern (arrow). **(E)** Delayed phase contrast-enhanced T1WI reveals persistent enhancement with notable enhancement in previously necrotic areas (arrow pointing to delayed enhancement within necrotic/cystic regions that decrease in size). **(F)** DWI displays hyperintensity of the tumor (arrow), indicating restricted diffusion. **(H)** ADC map shows corresponding hypointensity (arrow), comparable to the spleen in the same plane, confirming true diffusion restriction. **(I)** Hematoxylin and eosin staining (HE) at 400× magnification reveals tumor cells with pale or eosinophilic cytoplasm (arrow pointing to characteristic tumor cells), large oval nuclei containing vesicular chromatin, and visible eosinophilic nucleoli, set against a background rich in lymphocytes and plasma cells. **(J)** EBER staining at 400×magnification demonstrates positive EBER expression in the tumor cells (arrow indicating positive nuclear staining), confirming EBV infection.

Among the 3 cases of hepatic EBV+ IFDCS, all underwent both non-contrast and contrast-enhanced MRI examinations. On T1WI, 1 case exhibited a slightly hyperintense signal, while 2 cases demonstrated slightly hypointense signals. On T2WI, all cases revealed either homogeneous or heterogeneous slightly hyperintense signals. The 2 cases (with diameters of 6.2 cm and 2.5 cm) showed a heterogeneous enhanced filling-in pattern, remaining slightly hypointense in the delayed phase. The 1 case (diameter 1.9 cm) exhibited an enhancement pattern similar to that observed on CT, characterized by a rapid wash-in and slow wash-out pattern, with significant enhancement in the arterial phase, decreasing in the venous and delayed phases but remaining slightly hyperintense in the delayed phase. In 1 case (diameter 6.2 cm), the areas of necrosis and cystic degeneration showed a reduction in enhancing areas and partial enhancement in the delayed phase (as shown in **Figure 2**). The tumor capsules exhibited delayed enhancement, and no fat signals were detected within the tumors. On DWI, the solid components exhibited high signal intensity, while on ADC maps, they appeared as low signal intensity.

The ADC values of the solid portions of the 16 hepatic and splenic EBV+ IFDCS lesions did not show a statistically significant difference when compared to the ADC values of the corresponding normal spleen tissue, with a *P* > 0.05 (*P* = 0.793) (**Table 3**).

**Table 3:** Comparison of ADC values between lesions and normal spleen in 16 patients (× 10^-3^ mm^2^/S).

Variables	Median *M* (*P_25_*, *P*_75_)	Mann-Whitney U test between two groups	
		*Z* value	*P* value
Lesion ADC value	0.927 (0.591,0.927)	-0.263	0.793
Normal spleen ADC value	0.829 (0.581,0.829)		

Metastasis and recurrence: Follow-up data for the 16 patients in the cohort, for a duration of 1 to 60 months, revealed no instances of recurrence or metastasis.

### 3.3 Pathological findings

All 16 lesions were surgically excised and subsequently analyzed. The pathological features of EBV+ IFDCS exhibited well-defined tumor boundaries, presenting as grayish-white or grayish-yellow masses. Histological examination confirmed the presence of a fibrous pseudocapsule, within which small blood vessels were identified. Tumor cells were arranged in short spindle-shaped, interlacing, and whorl-like patterns, accompanied by extensive infiltration of lymphocytes and plasma cells in the stroma. Areas of necrosis and cystic degeneration were noted, and some cases exhibited scattered epithelioid granulomatous nodules and vascular proliferation. Immunohistochemistry (IHC) analysis of the 16 lesions demonstrated positive expression of 3 to 5 markers among CD3, CD21, CD23, CD35, Vimentin, and SMA. Ki-67 positivity ranged from 10% to 40%. EBER was positive in all EBV+ IFDCS lesions, and IgG4 positivity was observed in 5 lesions.

## 4 Discussion

EBV+ IFDCS is a rare, low-grade malignant tumor. To date, most reported imaging findings have been derived from individual cases or small cohorts. This study represents the largest imaging-pathological correlation analysis of hepatic and splenic EBV+ IFDCS cases to our knowledge.

In 1986, Monda et al. reported the first four cases of tumors originating from FDCS (2). Subsequently, in 2001, Cheuk et al. documented 11 cases occurring in the abdominal cavity, which exhibited distinctive morphological features characterized by a lymphocyte- and plasma cell-rich background, closely resembling idiopathic plasmacytoma-like follicular dendritic cell sarcoma (IPT-like FDCS) (3). This led to their classification as IPT-like FDCS. The tumor cells exhibited positive reactions to antibodies related to FDC markers, confirming their FDC origin.

Recent studies have established a link between this tumor type and EBV infection. EBV+ IFDCS of the liver and spleen may represent a clonal proliferation of EBV-infected follicular dendritic cells. The presence of EBV within the tumor population is typically showed through *in situ* hybridization (ISH) for EBER or through IHC staining for latent membrane protein, both of which are essential for the diagnosis of EBV+ IFDCS (6). Consequently, the fifth edition of the WHO classification has renamed it EBV+ IFDCS.

### 4.1 Clinical manifestations

Building upon the brief clinical overview provided in the introduction, our case series offers several important observations that both confirm and expand upon previously reported clinical characteristics of EBV+ IFDCS. Hepatosplenic EBV+ IFDCS is more prevalent in adults and occurs more frequently in female patients compared to classic follicular dendritic cell sarcoma (7–9). In our case series, the male-to-female ratio was 1:2.2, indicating a female predominance, which aligns with previous studies. Research suggests a significantly higher incidence of this disease among Asian populations, pointing to potential racial susceptibility (10).

In our study, we observed statistical differences in the age of onset and tumor diameter between hepatic and splenic EBV+ IFDCS. Specifically, splenic EBV+ IFDCS presented with an older age of onset and a larger tumor diameter compared to its hepatic counterpart. This difference may be influenced by the small sample size of hepatic EBV+ IFDCS cases.

Most patients in our series were either asymptomatic or presented with non-specific clinical symptoms. Notably, only one case of splenic EBV+ IFDCS presented with hematochezia, which was attributes to thrombocytopenia and resolved following splenectomy. A previous report in the literature documented a similar case associated with thrombocytopenia, although no direct link between thrombocytopenia and this tumor has been established (11). The remaining 15 cases were identified during routine physical examinations, with one case exhibiting significant growth over a one-year follow-up period. In instances involving larger tumors, patients may experience abdominal distension, abdominal pain, or splenomegaly, while fever may occur occasionally.

### 4.2 Imaging and pathological features

Histologically, follicular dendritic cell sarcoma is primarily classified into two types: (1) Conventional follicular dendritic cell sarcoma, characterized by tumor cells arranged in layers, bundles, and diffuse patterns, accompanied by a small number of lymphocytes; and (2) EBV+ IFDCS, which consists mainly of scattered spindle or oval tumor cells with a significant presence of lymphocytes and plasma cells.

The pathological features observed in the cases included in this study are as follows: All lesions were solitary and had well-defined margins from adjacent structures. A fibrous capsule was observed, containing small blood vessels, which underlies the delayed enhancement noted in the capsule on MRI. The lesions generally appeared as grayish-white to grayish-yellow masses. Tumor cells exhibited spindle-shaped or oval morphology, arranged in interlacing and whorl-like patterns, with significant cellular atypia and visible mitotic figures. The stroma exhibited extensive infiltration by lymphocytes and plasma cells, accompanied by vascular proliferation, cystic degeneration, and necrosis.

In terms of IHC, the tumors commonly expressed one or more follicular dendritic cell markers (CD21, CD23, and CD35) to varying extents. All cases tested positive for EBER through *in situ* hybridization. Among the 16 cases of EBV+ IFDCS examined in this study, 5 cases (31%) were found to be positive for IgG4, suggesting the necessity for further research into the association between EBV+ IFDCS and IgG4-related sclerosing disease.

The CT imaging characteristics of the 14 cases of hepatic and splenic lesions in this study are summarized as follows: Most tumors were asymptomatic and detected incidentally during routine physical examinations. Calcification was occasionally noted, with only one case exhibiting peripheral encapsulated calcification. Necrosis and cystic degeneration were common in larger tumors. Among the 5 cases (31%) with tumor diameters ranging from 1.9 cm to 4.9 cm, no central necrosis or cystic degeneration was observed. In contrast, the 9 cases (69%) with diameters between 3.6 cm and 19 cm displayed areas of necrosis. Of these, 4 cases showed no changes in the extent of necrosis and cystic degeneration in the venous phase following enhancement, which pathology confirmed as liquefactive necrosis. In 5 cases, the areas of cystic degeneration and necrosis diminished in the venous phase compared to the arterial phase, which pathology identified as incomplete coagulative necrosis.

On CT, areas of cystic degeneration and necrosis showed no significant delayed enhancement. On non-contrast scans and delayed phases, 13 cases (93%) appeared iso- or hypodense. We speculate that this phenomenon may be attributed to slow tumor growth, sparse tumor cell distribution, and adequate blood supply. The 13 cases of hepatic and splenic EBV+ IFDCS displayed an enhanced filling-in pattern, with mild to moderate enhancement in the arterial phase. Additionally, 4 cases demonstrated prominent enhanced chaotic vascular shadows in the CT arterial phase. The lesions continued to enhance in the portal venous and delayed phases, likely related to the presence of small vessels within the tumor. Delayed enhancement is believed to be associated with extensive inflammatory cell infiltration, high cellular density, and potentially the lymphocyte-rich stroma (12).

One case of hepatic EBV+ IFDCS (diameter 1.9 cm) exhibited a rapid wash-in and slow wash-out enhancement pattern, differing from other hepatic and splenic EBV+ IFDCS cases. This variation is likely attributable to the small size of the lesion, dense vascular distribution, and the absence of necrosis or cystic degeneration. The inconsistent enhancement pattern observed in the liver aligns with findings from previous studies (13).

The MRI characteristics of the lesions can be summarized as follows:

1. All lesions were solitary with well-defined margins. On T1WI, they exhibited heterogeneous iso- to hypointense signals, while on T2WI, they appeared heterogeneously iso- to hyperintense. The signal intensity on T2WI varied depending on the proportion of inflammatory tissue, fibrous components, and necrotic areas within the tumor. Most lesions displayed a hypointense capsule on T2WI, which was pathologically confirmed as a fibrous capsule with capillaries, clearly delineated from surrounding tissues. This suggests a low-grade malignancy with expansive growth, providing a pathological basis for the delayed enhancement of the capsule on MRI (13). In larger tumors with incomplete peripheral capsules, invasiveness tended to increase.
2. The enhancement characteristics were largely consistent with those observed on CT. Only 2 cases exhibited enhancement in the central areas of cystic degeneration and necrosis during the delayed phase on MRI, a feature not apparent on the corresponding CT images. This discrepancy may be attributed to the longer delayed phase in MRI (5–6 minutes) compared to CT (85–90 seconds). In cases of incomplete necrosis, the presence of fibrous components and proliferating small blood vessels can lead to delayed enhancement. Previous literature has reported only one case displaying this feature, highlighting MRI’s advantage over CT in illustrating this rare enhancement pattern (14).
3. Lesions appeared hyperintense on DWI, with ADC values similar to those of normal spleen tissue. This may be related to the dense arrangement of lymphocytes and plasma cells within the tumor, which restricts water molecule diffusion, analogous to the lymphocyte-rich composition of the spleen. Consequently, DWI and ADC mapping in MRI hold significant diagnostic value for EBV+ IFDCS. MRI provides a significant advantage in visualizing the capsule, as illustrated in **Figures 2C, E** (arrows). In this study, 10 out of 12 MRI images (83%) distinctly revealed the capsule structure with delayed enhancement, whereas only 9 of the 14 CT images (64%) showed a hypodense capsule structure. This discrepancy may be attributed to the low contrast between the capsule density and the surrounding liver or spleen parenchyma. The delayed phase for MRI lasts between 5 to 6 minutes, considerably longer than that of CT. This extended duration allows for enhanced observation of the capsule’s delayed enhancement, attributed to its small blood vessels. Consequently, MRI is superior to CT in assessing the peripheral capsule structure of EBV+ IFDCS.

Both CT and MRI effectively reveal components of necrosis and cystic degeneration within the lesions. However, MRI excels in illustrating the reduction in enhancement area and notable delayed enhancement in regions of cystic degeneration and necrosis.

Hepatosplenic EBV+ IFDCS predominantly presents as solitary lesions. In the existing English literature, only two cases of splenic EBV+ IFDCS have been documented as multiple lesions (7, 15). Currently, there is no evidence to suggest that the presence of multiple lesions impacts the prognosis of this disease. Typically, multiple lesions are smaller and exhibit a lower incidence of necrosis, which can increase the likelihood of misdiagnosis.

### 4.3 Recurrence and/or metastasis

Among the 16 cases of hepatic and splenic EBV+ IFDCS analyzed in this study, none exhibited metastasis or recurrence during the follow-up period. This finding aligns with most existing research, which supports the notion that EBV+ IFDCS is a low-grade malignant tumor (16). However, some studies have indicated that EBV+ IFDCS can recur or metastasize to the liver and lungs, or abdominal cavity (7). Wu et al. followed 57 patients diagnosed with hepatic and splenic EBV+ IFDCS and found that 9 cases (15.8%) experienced local and/or distant recurrence following initial treatment (9). The 1-year and 5-year progression-free survival rates for the entire cohort were 91.5% and 56.1%, respectively.

Currently, surgical resection remains the primary treatment modality, as adjuvant chemotherapy and radiotherapy have not showed improvements in overall survival or disease-free survival. According to the literature, factors associated with poor prognosis for this tumor include younger patient age, larger tumor size (≥ 6 cm), presence of necrosis, frequent mitotic figures (≥ 5 per high-power field), and notable histological atypia (17). The primary sites of metastasis for this tumor include the lungs, liver, lymph nodes, and bones. However, due to the limited number of reported cases in the literature, the precise factors influencing prognosis require further validation through the accumulation of additional cases and extended follow-up periods. Therefore, complete surgical resection is essential, and it is recommended that physicians thoroughly examine the resected specimens to confirm clear margins.

### 4.4 Differential diagnosis

Splenic EBV+ IFDCS must be differentiated from the following lesions: (1) Sclerosing angiomatoid nodular transformation of the spleen usually appears as a solitary mass with low signal intensity on T2WI (unlike the intermediate-to-high signal in EBV+ IFDCS) and demonstrates centripetal progressive enhancement, nodular enhancement, and spoke-wheel enhancement. For a comprehensive comparison of imaging features, readers are referred to **Supplementary Table S1** (18). (2) Splenic lymphoma typically presents as multiple lesions that may coalesce into masses (whereas EBV+ IFDCS is usually solitary), showing mild homogeneous enhancement (in contrast to the peripheral enhancement pattern of EBV+ IFDCS) and high signal on DWI (19). (3) Splenic angiosarcoma: This highly invasive tumor often lacks a capsule and presents with ill-defined margins. It typically shows central patchy enhancement with persistent filling (20).

Hepatic EBV+ IFDCS must be differentiated from the following conditions: (1) Primary hepatocellular carcinoma: This tumor often presents with a history of cirrhosis and typically shows enhancement in the arterial phase, followed by washout in the portal venous and delayed phases. Smaller lesions of hepatic EBV+ IFDCS may appear slightly hyperdense in the portal venous and delayed phases, aiding in their differentiation. (2) Hepatic cavernous hemangioma: This lesion exhibits a progressively enhancing filling-in pattern, generally without areas of necrosis or cystic degeneration, and appears hyperdense or hyperintense in the delayed phase.

### 4.5 Study limitations and future directions

This study has several limitations. First, due to its retrospective and multi-institutional nature, standardized semi-quantitative assessment of EBER expression intensity was not performed, preventing correlation of EBV infection degree with imaging characteristics. Second, EBV serologic studies were not systematically conducted across all three institutions. Third, the relatively small sample size, although one of the largest imaging series of this rare entity, limits statistical power. To address these limitations, we plan to conduct a multi-center prospective study with standardized protocols for EBER expression scoring and EBV serologic testing to better elucidate the relationship between viral factors and radiological-pathological features of this rare entity. This future study will implement standardized protocols across participating institutions, including semi-quantitative scoring of EBER expression intensity, comprehensive EBV serologic testing, and correlation of these viral parameters with detailed imaging characteristics. We anticipate that this prospective approach will provide more robust evidence regarding how the degree of EBV infection influences tumor necrosis and other imaging features, potentially enhancing the diagnostic accuracy for this rare entity.

## 5 Conclusion

In conclusion, hepatosplenic EBV+ IFDCS is a rare entity with distinctive imaging characteristics. These tumors typically appear as hypodense masses on CT and show slight low signal intensity on T1WI with slight high signal intensity on T2WI MRI sequences. They demonstrate mild to moderate enhancement on contrast-enhanced CT and MRI, with characteristic hypointensity on delayed phases. Tumor capsules are better visualized on MRI than CT, particularly on T2WI and contrast-enhanced sequences. Cystic degeneration and necrosis appear as non-enhancing areas on both modalities, though more clearly defined on MRI. Most lesions are solitary, well-defined masses with homogeneous or heterogeneous appearance depending on the presence of hemorrhage or necrosis. MRI provides superior soft-tissue contrast and is more sensitive for detecting capsules and internal characteristics. These imaging features, combined with pathological confirmation showing EBV positivity, are essential for accurate diagnosis of this rare malignancy. However, as an exploratory retrospective analysis, this study describes the imaging and pathological features of EBV+ IFDCS, but due to study design limitations, definitive radiologic-pathologic correlations cannot yet be established. This remains to be explored in future prospective studies.

## Data Availability

The original contributions presented in the study are included in the article/**Supplementary Material**. Further inquiries can be directed to the corresponding authors.
