# Supplementary material for: Imaging and pathological characteristics of hepatosplenic EBV-positive inflammatory follicular dendritic cell sarcoma
Source: Front Oncol. 2025 May 16;15:1552193. doi: 10.3389/fonc.2025.1552193 (PMC12122307; doi:10.3389/fonc.2025.1552193)
Supplement: Supplementary file 1 [file Table1.docx]

Table S1: Comparison of key imaging features between EBV+ IFDCS and its main differential diagnoses.

| **Disease** | **T1WI signal** | **T2WI signal** | **DWI signal** | **Enhancement pattern** | **Borders** | **Multiplicity** | **Additional features** |
| --- | --- | --- | --- | --- | --- | --- | --- |
| **EBV+ IFDCS (Spleen)** | Hypointense | Intermediate to hyperintense | Mild-moderate restriction | Peripheral enhancement with central hypointensity | Well-defined | Usually solitary | Subcapsular location common |
| **SANT** | Hypointense | Hypointense | Mild restriction | Spoke-wheel, centripetal progressive enhancement | Well-defined | Usually solitary | Central stellate scar |
| **Splenic lymphoma** | Hypointense | Intermediate to hyperintense | Marked restriction | Mild homogeneous enhancement | Variable definition | Often multiple | May involve other organs |
| **Splenic angiosarcoma** | Heterogeneous hypointense | Heterogeneous, hyperintense | Variable restriction | Heterogeneous with vascular lakes | Ill-defined, invasive | Can be multifocal | Hemorrhage common |
| **EBV+ IFDCS (Liver)** | Hypointense | Intermediate to hyperintense | Mild-moderate restriction | Peripheral enhancement with central hypointensity | Well-defined | Usually solitary | Subcapsular location common |
| **Hepatocellular carcinoma** | Slightly hypointense | Slightly hyperintense | Moderate-marked restriction | Arterial enhancement with washout | Well-defined, often with capsule | Can be solitary or multiple | Background cirrhosis common |
| **Hepatic hemangioma** | Hypointense | Markedly hyperintense | No restriction (T2 shine-through) | Peripheral nodular with progressive fill-in | Well-defined | Often multiple | Very high T2 signal |
